# *Visinin‐like 1*, a novel target gene of the Wnt/β‐catenin signaling pathway, is involved in apoptosis resistance in colorectal cancer

**DOI:** 10.1002/cam4.5970

**Published:** 2023-04-25

**Authors:** Hiroki Tage, Kiyoshi Yamaguchi, Saya Nakagawa, So Kasuga, Kiyoko Takane, Yoichi Furukawa, Tsuneo Ikenoue

**Affiliations:** ^1^ Division of Clinical Genome Research, Advanced Clinical Research Center, Institute of Medical Science The University of Tokyo Tokyo Japan

**Keywords:** apoptosis, colorectal cancer, Visinin‐like 1 (VSNL1), Wnt/β‐catenin signaling

## Abstract

**Background:**

Abnormal activation of Wnt/β‐catenin signaling is associated with various aspects of cancer development. This study explored the roles of novel target genes of the Wnt/β‐catenin signaling pathway in cancer cells.

**Methods:**

Using the haploid chronic myelogenous leukemia cell line HAP1, RNA sequencing (RNA‐seq) was performed to identify genes whose expression was increased by *APC* disruption and reversed by β‐catenin knockdown (KD). The regulatory mechanism and function of one of the candidate genes was investigated in colorectal cancer (CRC) cells.

**Results:**

In total, 64 candidate genes whose expression was regulated by Wnt/β‐catenin signaling were identified. Of these candidate genes, the expression levels of six were reduced by β‐catenin KD in HCT116 CRC cells in our previous microarray. One of these genes was *Visinin‐like 1* (*VSNL1*), which belongs to the neuronal calcium‐sensor gene family. The expression of *VSNL1* was regulated by the β‐catenin/TCF7L2 complex via two TCF7L2‐binding elements in intron 1. VSNL1 KD‐induced apoptosis in VSNL1‐positive CRC cells. Additionally, forced expression of wild‐type VSNL1, but not a myristoylation, Ca^2+^‐binding, or dimerization‐defective mutant, suppressed the apoptosis induced by camptothecin and doxorubicin in VSNL1‐negative CRC cells.

**Conclusion:**

Our findings suggest that *VSNL1*, a novel target gene of the Wnt/β‐catenin signaling pathway, is associated with apoptosis resistance in CRC cells.

## 1 INTRODUCTION

The Wnt/β‐catenin signaling pathway, also known as the canonical Wnt signaling pathway, is a highly conserved mechanism that regulates embryonic development and adult stem cell maintenance.^1^ Deregulation of this pathway is associated with cancer development and progression.^2^

In normal embryonic development and stem cell maintenance, the binding of Wnt ligands to their receptors, Frizzled and lipoprotein receptor‐related protein (LRP), induces the association of AXIN with phosphorylated LRP. This removes AXIN from the cytoplasmic destruction complex (i.e., GSK3β, CK1, APC, and AXIN) leading to the stabilization of β‐catenin.^1^ Stabilized free β‐catenin translocates to the nucleus, where it forms a complex with TCF/LEF family transcription factors and induces the transcription of target genes. In normal adult cells, an intact destruction complex induces phosphorylation of β‐catenin at its N‐terminal Ser/Thr motif by CK1 and GSK3β, resulting in β‐catenin degradation via the ubiquitin/proteasome system.^1^

The frequency of Wnt/β‐catenin signaling activation varies among cancers. This signaling pathway is activated in more than 90% of cases of colorectal cancer (CRC), mainly because of a loss‐of‐function mutation or deletion of *APC*, partially an activating mutation in *CTNNB1*, or a loss‐of‐function mutation in *AXIN1/2*.^3^, ^4^ This pathway is also activated in almost 30% of cases of hepatocellular carcinoma, mainly because of a mutation in *CTNNB1* or a mutation or deletion of *AXIN1*.^4^, ^5^ Furthermore, the Wnt/β‐catenin signaling pathway is activated by the BCR‐ABL fusion protein produced by the reciprocal translocation t(9;22) (q34;11), which is found in most cases of chronic myelogenous leukemia (CML).^6^ The fusion protein phosphorylates two tyrosine residues (Tyr86 and Tyr654) in β‐catenin, leading to β‐catenin stabilization and inhibition of its degradation by the destruction complex.^6^

Multiple target genes of the Wnt/β‐catenin signaling pathway have been identified,^7^ including the oncogenes *CCND1* ^8^ and *MYC*,^9^ the stemness markers *LGR5* ^10^ and *CD44*,^11^ and the negative‐feedback regulator *AXIN2*.^12^, ^13^ However, some downstream targets have not been identified. Furthermore, additional novel target genes must be identified to improve the broader understanding of mechanisms that underlie Wnt/β‐catenin‐mediated carcinogenesis and to develop new treatments for tumors that involve activation of the signaling pathway.

Visinin‐like 1 (VSNL1) is a calcium‐sensor protein that belongs to a subfamily of neuronal calcium‐sensor proteins. It contains a myristoylation‐consensus sequence (M‐G‐X3‐S) in the N‐terminus and four EF‐hand motifs (EF1–4) that are important for membrane targeting and Ca^2+^‐binding, respectively. The interaction of VSNL1 with Ca^2+^ induces N‐terminal myristoylation and membrane translocation of VSNL1 by a mechanism known as the Ca^2+^‐myristoyl switch.^14^ EF2 and EF3 bind to Ca^2+^ in a cooperative manner.^15^ Additionally, Ca^2+^‐dependent VSNL1 dimerization enhances the membrane‐targeting Ca^2+^‐myristoyl switch function of VSNL1.^16^

Neuronal calcium‐sensor proteins have been evaluated for their effects on neurons because these proteins are expressed predominantly in nervous system tissues, such as the brain. However, VSNL1 is also expressed in the heart, liver, lung, testes, skin, and colon,^14^ and so the role of VSNL1 in non‐neuronal tissues has recently been gaining attention.

The role of VSNL1 in tumorigenesis is reportedly cancer type dependent. It inhibits the migration and invasion of squamous cell carcinoma,^17^, ^18^, ^19^ whereas it promotes the proliferation, migration, and invasion of gastric cancer and neuroblastoma.^20^, ^21^ VSNL1 reportedly promotes CRC proliferation, migration, and invasion.^22^

In the present study, we identified *VSNL1* as a novel target gene of the Wnt/β‐catenin signaling pathway. We also found that Wnt/β‐catenin‐dependent *VSNL1* expression is mediated via TCF7L2/β‐catenin‐binding elements in intron 1 of *VSNL1*. Finally, we demonstrated that VSNL1 is involved in apoptosis resistance in CRC cells.

## 2 MATERIALS AND METHODS

### 2.1 Cell lines

The human CRC cell lines HCT116, LoVo, LS174T, RKO, SW48, and SW480 were obtained from American Type Culture Collection. The human CML cell line HAP1 was purchased from Horizon Discovery. An embryonic kidney cell line, 293FT, was purchased from Thermo Fisher Scientific. The Platinum‐A (PLAT‐A) retroviral packaging cell line was a kind gift from Dr. Toshio Kitamura (The University of Tokyo).

All cell lines were cultured in appropriate media (McCoy's 5A Medium Modified for HCT116; F‐12 K for LoVo; Eagle's minimum essential medium for LS174T; Dulbecco's modified Eagle medium for RKO, 293FT, and PLAT‐A; Leibovitz's L‐15 for SW48 and SW480; and Iscove's modified Dulbecco's medium for HAP1) supplemented with 10% fetal bovine serum and antibiotic‐antimycotic solution (Wako Pure Chemical) at 37°C.

### 2.2 CRISPR/Cas9‐mediated gene editing

Guide RNAs against *APC* were designed using CRISPRdirect software (https://crispr.dbcls.jp/). The two gRNAs used in this study are shown in Table S1. Double‐stranded DNA with 4‐bp overhangs at both ends was cloned into the *Bbs*I site in the pSpCas9(BB)‐2A‐GFP vector (PX458; Addgene). HAP1 cells were transfected with PX458‐*APC*‐gRNA using FuGENE6 (Promega). After 24 h, green fluorescent protein‐positive cells were isolated using fluorescence‐activated cell sorting (FACSAria; BD Biosciences). Subsequently, single‐cell cloning was performed and clones without APC expression were identified by Western blotting. Additionally, *APC* gene disruption at the target locus in APC‐knockout (KO) clones was confirmed by TA cloning (Thermo Fisher Scientific) and Sanger sequencing using the 3500xL Genetic Analyzer (Thermo Fisher Scientific).

### 2.3 Gene silencing

Two independent siRNAs targeting *VSNL1* (hs.Ri.VSNL1.13.2; siVSNL1 #2 and hs.Ri.VSNL1.13.3; siVSNL1 #3) were purchased from Integrated DNA Technologies. Two siRNAs targeting *CTNNB1* and control siRNA were used in our previous study.^23^ The target sequences of the siRNAs are listed in Table S1. Cells were plated 1 day before transfection with 20 nM siRNA using Lipofectamine RNAiMAX (Thermo Fisher Scientific). Total RNA was extracted 48 h later using the RNeasy Plus Mini Kit (Qiagen) according to the manufacturer's protocol. The silencing effects of the siRNAs were evaluated by RT‐qPCR or Western blotting.

### 2.4 RNA‐seq analysis

Total RNA was isolated from parental HAP1 cells, HAP1‐APC‐KO cells (clones #2 and #3), and HAP1‐APC‐KO cells (clone #2) treated with *CTNNB1* siRNA or control siRNA, as described previously.^24^ Sequence libraries were prepared using 100 ng of total RNA and an Ion AmpliSeq Transcriptome Human Gene Expression Kit (Thermo Fisher Scientific) according to the manufacturer's instructions. Sequencing of the libraries was performed on the Ion Proton system using an Ion PI Hi‐Q Sequencing 200 Kit along with Ion PI Chip Kit v3 (Thermo Fisher Scientific). Raw sequencing reads were mapped to hg19_AmpliSeq_Transcriptome_ERCC_v1 by the Torrent Mapping Alignment Program. The data were analyzed using the AmpliSeqRNA plug‐in v5.2.0.3 in the Torrent Suite Software v5.2.2 (Thermo Fisher Scientific). Data processing was carried out with GeneSpring GX13.1 (Agilent Technologies). The expression levels of genes with Benjamini–Hochberg‐corrected *p*‐values <0.05 were determined to be significantly altered. Kyoto Encyclopedia of Genes and Genomes (KEGG) enrichment pathway analysis of differentially expressed genes was performed using the Molecular Signatures Database (MSigDB) v7.5.1 (http://www.broadinstitute.org/gsea/msigdb/index.jsp).

### 2.5 Reporter plasmids and luciferase assay

To construct a reporter plasmid for the candidate promoter region (CPR) and candidate enhancer region (CER) of *VSNL1*, the 5′‐flanking 1.2‐kb region (hg19‐chr2: 17721005‐17722174) and a 253‐bp region within *VSNL1* intron 1 (hg19‐chr2: 17760951‐17761203) were amplified using primer sets (Table S1) and BAC clone RP11‐622C4 (Advanced GenoTechs, Tsukuba, Japan) as a template. The amplicons were digested using *Xho*I and *Bgl*II, then subcloned into pGL4.14 and pGL4.23 luciferase reporter vectors (Promega), respectively. For the construction of mutant reporter plasmids with substitutions from AA to GC in putative TCF7L2‐binding sites, site‐directed mutagenesis was carried out using the primers in Table S1.

A reporter assay was performed to identify the Wnt/β‐catenin‐dependent regulatory region of *VSNL1* in CRC cells. The cells seeded on 12‐well plates were transfected with 1 μg of luciferase reporter plasmid and 0.05 μg of pRL‐null (Promega), a *Renilla* luciferase plasmid, in combination with *CTNNB1* siRNAs (siCTNNB1 #9 and #10) or control siRNA at a concentration of 20 nM, or in combination with 1 μg of pCAGGS or pCAGGS‐dominant negative TCF7L2 (dnTCF7L2) using Lipofectamine 2000 (Thermo Fisher Scientific), then incubated for 48 h. To determine the transcriptional activity of β‐catenin/TCF7L2 complex in HAP1 cells, the TOPFLASH/FOPFLASH (TOP/FOP) assay was performed, as previously described.^25^ After harvesting cells, a dual luciferase assay was performed using the PicaGene Dual Luciferase Assay System according to the manufacturer's instructions (TOYO B‐Net, Tokyo, Japan).

### 2.6 RT‐qPCR

One microgram of total RNA was reverse‐transcribed using ReverTra Ace (TOYOBO). qPCR was carried out using the KAPA SYBR Fast qPCR Kit (Kapa Biosystems) and the StepOnePlus System (Thermo Fisher Scientific). The primer sequences for qPCR are listed in Table S1. *Glyceraldehyde‐3‐phosphate dehydrogenase* (*GAPDH*) was used as the control.

### 2.7 Western blotting

Western blotting was performed as described previously^23^ using the following primary antibodies: anti‐APC (sc‐895; Santa Cruz Biotechnology), anti‐AXIN2 (2151; Cell Signaling Technology), anti‐β‐catenin (9582; Cell Signaling Technology), anti‐VSNL1 (49468; Cell Signaling Technology), anti‐PARP (9532; Cell Signaling Technology), anti‐cleaved PARP (9541; Cell Signaling Technology), anti‐cleaved caspase‐3 (9664; Cell Signaling Technology), anti‐caspase‐8 (9746; Cell Signaling Technology), anti‐cleaved caspase‐9 (9505; Cell Signaling Technology), and anti‐β‐actin (A5441, Sigma‐Aldrich). All antibodies except anti‐β‐actin (1:3000) were diluted at 1:1000. Horseradish peroxidase‐conjugated goat anti‐mouse or anti‐rabbit IgG antibodies (GE Healthcare) were used as the secondary antibodies. Signals were quantified using ImageJ software (https://imagej.nih.gov/ij/).

### 2.8 Chromatin immunoprecipitation (ChIP)‐qPCR assay

HCT116 cells were cross‐linked with 1% formaldehyde for 10 min at room temperature (RT) and subsequently digested with micrococcal nuclease as described previously.^26^ Immunoprecipitation of protein‐DNA complexes was performed using 3 μg of anti‐TCF7L2 monoclonal antibody (6H5‐3; Millipore) or normal mouse IgG (negative control, Santa Cruz Biotechnology) bound to DynaBeads Protein G (Thermo Fisher Scientific). Co‐immunoprecipitated DNA fragments were purified using the conventional DNA extraction method, and analyzed by qPCR. The amplified *RNF43* enhancer (hg19‐chr17: 56472207‐56472326) was used as the positive control.^26^ The primer sequences for qPCR are listed in Table S1.

### 2.9 Cell cycle analysis

SW48 and SW480 cells treated with *VSNL1* siRNA or control siRNA were fixed with cold 70% ethanol, treated with 2 mg/mL RNase A at 37°C for 30 min and incubated with 20 μg/mL propidium iodide in phosphate‐buffered saline at RT for 30 min. After filtering through a nylon mesh, the cells were subjected to flow cytometric analysis using the BD FACSCalibur (BD Biosciences).

### 2.10 Retroviral transduction

The pMXs‐puro retroviral vector (a gift from Dr. Toshio Kitamura, The University of Tokyo) carrying *VSNL1* cDNA was constructed by amplifying the full‐length *VSNL1* (NM_003385.5) and inserting the amplicon into the vector. The sequences of primers for the amplification are presented in Table S1. *VSNL1* mutant vectors (defective for myristoylation [G2A], Ca^2+^‐binding [D73A/D109A], and dimerization [I136G/M137G]) were constructed via site‐directed mutagenesis using the primers listed in Table S1. Retroviral particles were produced by the transfection of pMXs‐puro (Mock) or pMXs‐VSNL1 constructs into PLAT‐A cells as described previously.^23^ RKO cells were infected with the retroviruses and selected using the medium that contained 1.2 μg/mL puromycin (Sigma‐Aldrich).

### 2.11 Cell proliferation assay

Proliferation of mock and wild‐type VSNL1‐expressing RKO cells were determined using the Cell‐Counting Kit‐8 according to the manufacturer's protocol (Dojindo).

### 2.12 Induction of apoptosis by anticancer drugs

RKO cells expressing wild‐type VSNL1 or its mutants were cultured in medium containing camptothecin (CPT; Wako Pure Chemical) or doxorubicin (DOX; LC Laboratories) at the indicated concentrations. After 48 h, total protein was extracted from the cells and apoptosis induction was evaluated via Western blotting for cleaved PARP.

### 2.13 Statistical analysis

Unpaired Student's *t*‐tests were applied to the RT‐qPCR and reporter assay data. Error bars are standard errors (SEs). For the statistical analysis of RNA‐seq data, we used Student's *t*‐test with Benjamini–Hochberg correction.

## 3 RESULTS

### 3.1 Disruption of the *APC* gene in HAP1 cells

To understand the role of the Wnt/β‐catenin signaling pathway in tumorigenesis, we explored novel target genes of the Wnt/β‐catenin signaling pathway using the CML cell line HAP1, which has a haploid genome and the *BCR*‐*ABL* fusion gene. We used the CRISPR‐Cas9 system to establish HAP1 cells in which the Wnt/β‐catenin signaling pathway was activated by *APC* gene disruption. Two guide RNAs (gRNAs) for the *APC* gene were used in the present study. Thus, three APC‐KO HAP1 clones were constructed (HAP1‐APC‐KO#1–3). Sanger sequencing revealed a 3‐bp in‐frame deletion (c.del318_320) in clone #1, a 2‐bp frameshift deletion (c.del2569_2570) in clone #2, and a 19‐bp frameshift deletion (c.del2558_2576) in clone #3 (Figure S1). The TOP/FOP reporter assay demonstrated higher TOP/FOP activity in the three *APC*‐KO clones, compared with the parental HAP1 cells (Figure S1). Furthermore, the mRNA expression level of *AXIN2*, a representative target gene of the Wnt/β‐catenin signaling pathway, was increased in HAP1‐APC‐KO clones and significantly suppressed by β‐catenin knockdown (KD) (Figure S1). Because the results of the TOP/FOP reporter assay suggested that Wnt/β‐catenin signaling activity was significantly greater in clones #2 and #3 than in clone #1 (Figure S1), clones #2 and #3 were used as HAP1‐APC‐KO cells in further analyses.

### 3.2 Identification of *VSNL1* as a novel target gene of the Wnt/β‐catenin signaling pathway

**FIGURE 1:**
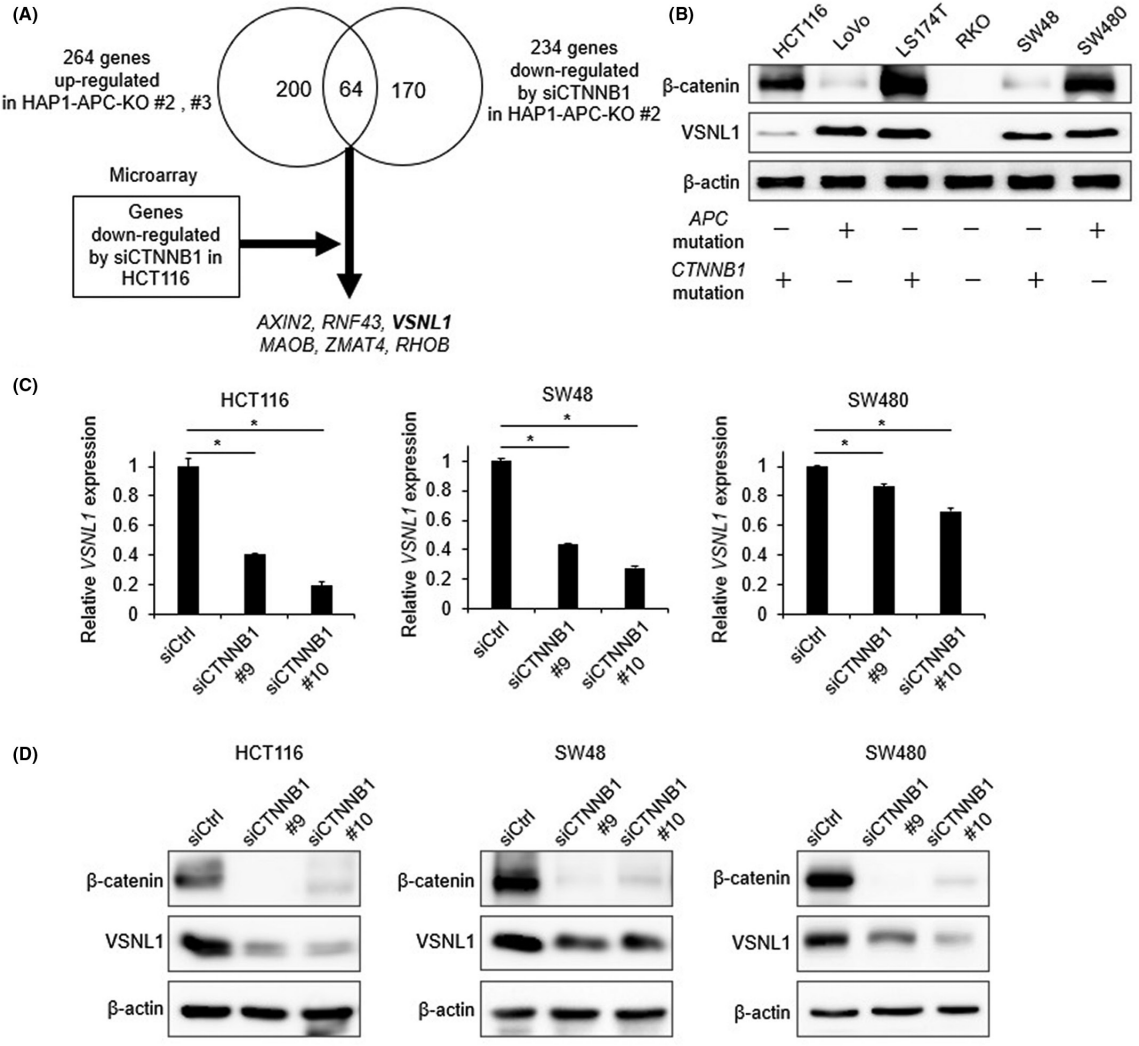
Identification of *VSNL1* as a novel Wnt/β‐catenin target gene in CRC cells. (A) Strategy for the identification of candidate target genes of the Wnt/β‐catenin signaling pathway. (B) Protein expression levels of VSNL1 and β‐catenin, and mutation statuses of *APC* and *CTNNB1*, in six CRC cell lines. (C) Relative expression levels of *VSNL1* mRNA in CRC cell lines (HCT116, SW48, and SW480) treated with control siRNA (siCtrl) and two *CTNNB1* siRNAs (siCTNNB1 #9 and #10) for 48 h. Data are presented as means ± SEs of ≥3 independent experiments. (D) Protein expression levels of VSNL1 and β‐catenin in CRC cell lines (HCT116, SW48, and SW480) treated with control siRNA (siCtrl) and two *CTNNB1* siRNAs (siCTNNB1 #9 and #10) for 48 h.

To identify novel target genes of the Wnt/β‐catenin signaling pathway, we performed RNA‐seq of parental HAP1, HAP1‐APC‐KO #2 and #3, and HAP1‐APC‐KO #2 cells treated with *CTNNB1* siRNA or control siRNA. We identified 264 genes whose expression was increased by twofold because of *APC* disruption in HAP1 cells and 234 genes for which expression was decreased to less than half by β‐catenin KD in HAP1‐APC‐KO cells (Figure 1A). The integration of these data revealed 64 candidate target genes of the Wnt/β‐catenin signaling pathway (Figure 1A, Table S2).

Previously reported target genes of the Wnt/β‐catenin signaling pathway, including *APCDD1*,^27^ *TNFRSF19*,^28^, ^29^ *RNF43*,^26^, ^30^ *AXIN2*,^12^, ^13^, ^31^ *NKD1*,^31^ *AHR*,^32^ *BAMBI*,^33^ and *MSX2*,^34^ were included in the 64 candidate genes (Table S2). KEGG pathway analysis of the 64 candidate genes using curated gene sets in the MSigDB showed that the “Wnt signaling pathway” was the only pathway whose associated genes were significantly enriched (Table S3). These results suggest the validity of our screening strategy.

To reduce the number of candidate genes regulated by the Wnt/β‐catenin signaling pathway, we integrated the microarray data of HCT116 CRC cells treated with *CTNNB1* and control siRNAs. Of the 64 genes, we selected genes whose expression levels in cells treated with *CTNNB1* siRNA were decreased to less than half of the levels in cells treated with control siRNA. Therefore, six genes were identified as target genes of the Wnt/β‐catenin signaling pathway, including four novel candidates (*VSNL1*, *MAOB*, *ZMAT4*, and *RHOB*) and two known target genes (*AXIN2* and *RNF43*) (Figure 1A).

Because *VSNL1* showed the greatest fold reduction after β‐catenin KD in the HCT116 microarray data of the four genes, we focused on *VSNL1* in further experiments. To confirm that *VSNL1* is regulated by the Wnt/β‐catenin signaling pathway, we measured the expression levels of *VSNL1* mRNA and VSNL1 protein using reverse‐transcription quantitative PCR (RT‐qPCR) and Western blotting, respectively. *VSNL1* expression was upregulated by *APC*‐KO in HAP1 cells and downregulated by β‐catenin KD in HAP1‐APC‐KO cells (Figure S2A–D).

### 3.3 Wnt/β‐catenin signaling‐dependent *VSNL1* regulation in CRC cells

We next evaluated whether VSNL1 expression is regulated by the Wnt/β‐catenin signaling pathway in CRC cells. VSNL1 protein expression was observed in the CRC cell lines HCT116, LoVo, LS174T, SW48, and SW480, which exhibited increased transcriptional activities of the β‐catenin/TCF7L2 complex because of mutations in *APC* or *CTNNB1* (Figure 1B). VSNL1 protein expression was not detected in RKO cells, which lacked mutations in *APC* and *CTNNB1*. We also found reduced expression levels of *VSNL1* mRNA and VSNL1 protein after β‐catenin KD using RT‐qPCR and Western blotting, respectively (Figure 1C,D). Our findings suggest that *VSNL1* is a target gene of the Wnt/β‐catenin signaling pathway in CRC cells.

### 3.4 *VSNL1* expression in CRC and colon adenoma tissues

We investigated the *VSNL1* expression level in CRC and colon adenoma using The University of Alabama at Birmingham Cancer data analysis portal (UALCAN; http://ualcan.path.uab.edu),^35^ Gene Expression Profiling Interactive Analysis (GEPIA; http://gepia.cancer‐pku.cn),^36^ and Oncomine (https://www.oncomine.org).^37^ The *VSNL1* expression level was significantly enhanced in colon and rectal adenocarcinoma tissues (Figure S3A,B). No difference was observed in the *VSNL1* expression level according to tumor stage (Figure S3C). Additionally, *VSNL1* expression was significantly greater in colon adenoma tissues than in normal colorectal tissues (Figure S4).

We also examined correlations of gene expression levels between representative Wnt target genes and *VSNL1* using The Cancer Genome Atlas (TCGA) CRC datasets in the cBioportal database (https://www.cbioportal.org).^38^ The *VSNL1* expression level was modestly correlated with the expression levels of *LGR5* and *CD44* (Figure S5A,B), whereas no correlation was observed between the expression levels of *VSNL1* and *AXIN2* (Figure S5C).

### 3.5 Identification of the TCF7L2‐interacting region in intron 1 of *VSNL1*

**FIGURE 2:**
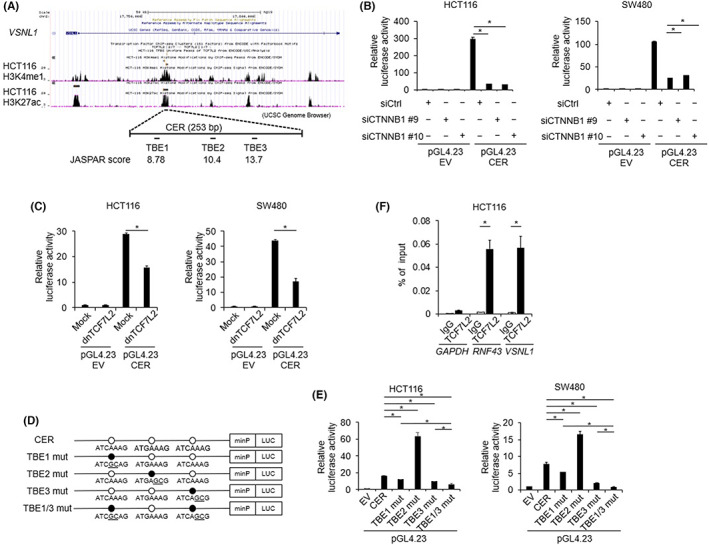
Regulation of *VSNL1* expression via TCF7L2‐binding elements in intron 1. (A) Genomic structure of *VSNL1* and the candidate TCF7L2‐binding elements in intron 1. (B) β‐catenin‐dependent transcriptional activity of the candidate enhancer region (CER). A reporter assay was performed using a reporter plasmid containing the CER (pGL4.23‐CER) or an empty vector (pGL4.23‐EV) in combination with the control siRNA or *CTNNB1* siRNA in HCT116 and SW480 cells. (C) TCF7L2‐dependent transcriptional activity of the CER. A reporter assay was performed using the reporter plasmids that contained the CER or empty vector in combination with a dominant‐negative TCF7L2 expression vector (dnTCF7L2) or an empty vector (Mock) in HCT116 and SW480 cells. (D) Interaction of the candidate region with TCF7L2 was analyzed by ChIP‐qPCR with anti‐TCF7L2 antibody. A TCF7L2‐binding region in *RNF43* was used as the positive control. (E) Schematic representation of the CER and mutant constructs of *VSNL1*. (F) Involvement of the three putative TCF7L2‐binding elements in the region with transcriptional activity. A reporter assay was performed using reporter plasmids with mutations in the putative TCF7L2‐binding elements. Data are presented as means ± SEs of ≥3 independent experiments.

We explored the candidate regulatory regions of *VSNL1* in the Wnt/β‐catenin signaling pathway using public databases. A search of the TCF7L2‐interacting peaks in the ChIP‐seq data of HCT116 in the Encyclopedia of DNA Elements (wgENCODEEH000629, ENCODE: http://www.encodeproject.org) revealed a peak (chr2:17,760,828‐17,761,203, GRCh37/hg19) in intron 1 of *VSNL1*. Furthermore, a canonical TCF7L2‐binding motif (chr2:17,761,125‐17,761,135) was detected in the same region. Therefore, we evaluated whether this region in intron 1 functions as a β‐catenin/TCF7L2‐mediated transcriptional enhancer. We performed a reporter assay using a plasmid containing a genomic region of 253 bp (chr2:17760951‐17761203) that included the identified canonical motif for TCF7L2 in intron 1 (Figure 2A). The reporter plasmid showed significantly greater activity compared with the activity of the empty vector in HCT116 and SW480 cells (Figure 2B). Importantly, the reporter activity was significantly suppressed by treatment with *CTNNB1* siRNA in both types of cells (Figure 2B). We also found that co‐transfection of a dominant‐negative form of TCF7L2 (dnTCF7L2) with the reporter plasmid containing the 253‐bp region significantly suppressed its reporter activity (Figure 2C). Next, we evaluated whether the Wnt/β‐catenin signaling pathway regulates *VSNL1* promoter activity. We generated a reporter plasmid containing an approximately 1.2‐kb region (−957 to +211 bp, from the transcription start site of *VSNL1*) and performed a reporter assay. Although we detected significantly greater reporter activity of the plasmid containing the region of interest compared with the activity of the empty vector in both HCT116 and SW480 cells, *CTNNB1* siRNA did not suppress the reporter activity (Figure S6A,B). These findings strongly suggest that the 253‐bp region in intron 1 is involved in the Wnt‐dependent transcriptional activation of *VSNL1*.

We also investigated the mechanism underlying the β‐catenin/TCF7L2‐dependent transcriptional activation of *VSNL1*. Using the JASPAR database, we identified three candidate TCF7L2‐binding elements (TBE1–3) in the 253‐bp region in intron 1 (Figure 2A). To determine the involvement of these elements in the β‐catenin/TCF7L2‐dependent transcriptional activation, we performed a reporter assay using the mutant reporter plasmids, along with the substitution of two nucleotides that disrupt the consensus TCF7L2‐binding sequence in each element (TBE1–3 mut) (Figure 2D). Compared with the activities of wild‐type plasmids, mutant reporter plasmids with substitution in TBE1 and TBE3 showed lower reporter activities in both HCT116 and SW480 cells (Figure 2E). In contrast, TBE2 mutant reporter plasmids showed higher reporter activities in both cell lines (Figure 2E). These results suggest TBE1 and TBE3 to be candidate enhancer regions of the β‐catenin/TCF‐dependent transcriptional activation of *VSNL1*, whereas TBE2 may act as a silencer element of *VSNL1* transcriptional activation. Moreover, a plasmid containing a combination of TBE1 and TBE3 mutations (TBE1/3 mut) showed further reduction in activity, compared with the activity of TBE1 mut or TBE3 mut (Figure 2E). Additionally, the reporter activity of TBE1/3 mut was not suppressed by *CTNNB1* siRNA (Figure S7). These findings suggest that TBE1 and TBE3 play a pivotal role in the β‐catenin/TCF‐dependent transcriptional activation of *VSNL1*.

To confirm the interaction of TCF7L2 with the candidate enhancer region of *VSNL1* in intron 1, we performed a ChIP‐qPCR assay using an anti‐TCF7L2 antibody and specific primer sets for the region. An *RNF43* region that interacts with TCF7L2 was used as the positive control. The DNA fragments that immunoprecipitated with the anti‐TCF7L2 antibody contained 49.3‐fold greater concentrations of the target region, compared with the DNA fragments that immunoprecipitated with the control IgG (Figure 2F), suggesting that TCF7L2 binds to the region in intron 1 of *VSNL1*.

### 3.6 Induction of apoptosis by VSNL1 KD in CRC cells

To investigate the function of VSNL1 in CRC cells, we performed *VSNL1* KD using two independent siRNAs. First, we examined whether VSNL1 affects Wnt/β‐catenin signaling activity in CRC cells. The results of TOP/FOP reporter assays using SW48 and SW480 cells, which carry *APC* and *CTNNB1* mutations, respectively, treated with two independent *VSNL1* siRNAs revealed that VSNL1 KD did not affect Wnt/β‐catenin signaling activity in CRC cells (Figure S8A).

**FIGURE 3:**
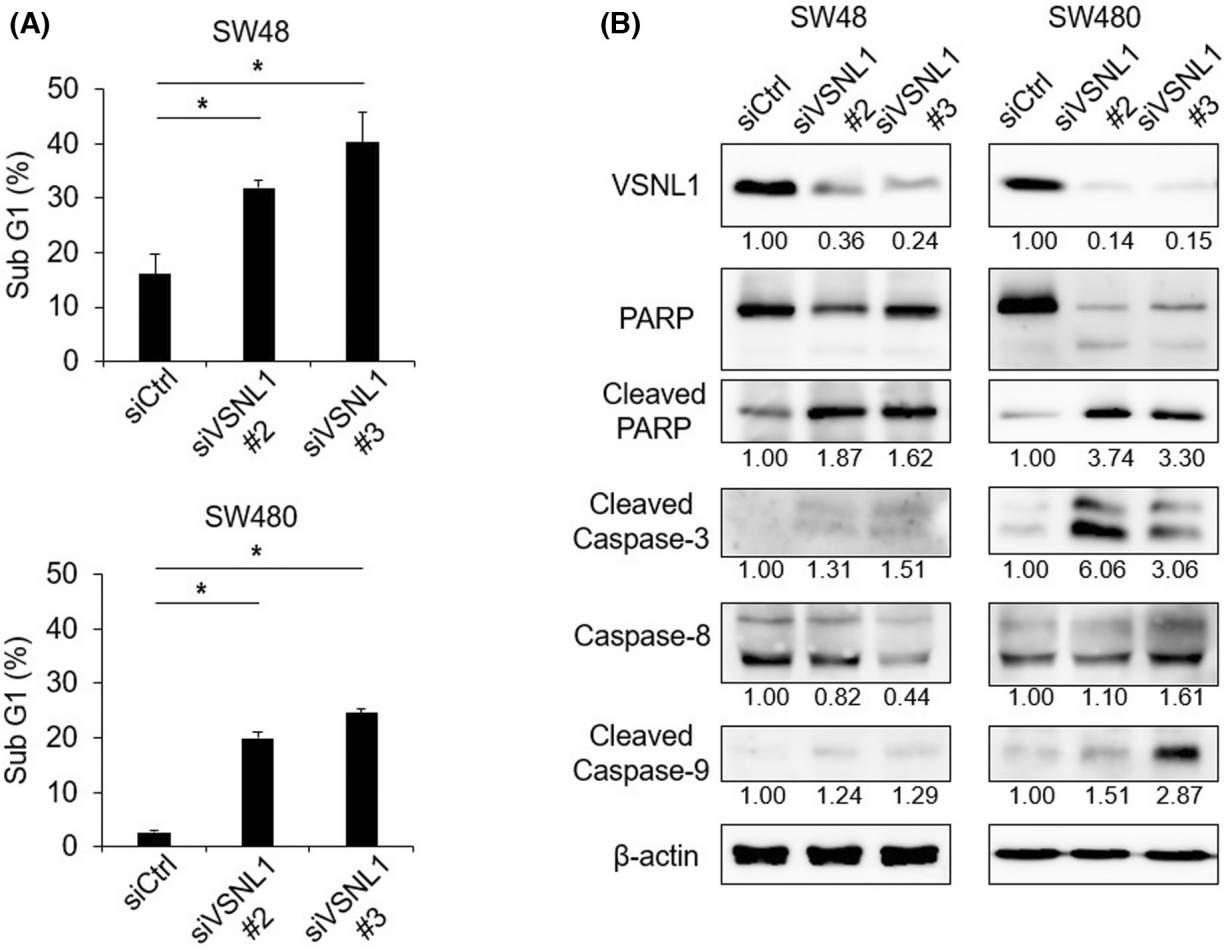
Induction of apoptosis by VSNL1 silencing in CRC cells. (A) SubG1 population of SW48 and SW480 cells treated with control siRNA or two *VSNL1* siRNAs (siVSNL1 #2 and #3). Data are presented as means ± SEs of ≥3 independent experiments. (B) Protein expression levels of cleaved PARP and cleaved caspase‐3 in SW48 and SW480 treated with control siRNA or two different *VSNL1* siRNAs (siVSNL1 #2 and #3). (B) Analysis of cleavage of PARP and caspase‐3, ‐8, and ‐9 in SW48 and SW480 cells treated with control siRNA or two *VSNL1* siRNAs (siVSNL1 #2 and #3). Band intensities were quantified using ImageJ and were normalized to β‐actin.

Because a greater number of floating cells was observed after treatment with siVSNL1 than after treatment with control siRNA, we next focused on apoptosis in further experiments. SW48 and SW480 cells were subjected to subG1 analysis. The subG1 fraction was increased by VSNL1 KD using two independent siRNAs in both cell lines (Figure 3A). Next, we performed Western blotting to examine the apoptosis markers cleaved PARP and cleaved caspase‐3. Both markers were induced by VSNL1 KD in both CRC cell lines (Figure 3B). Furthermore, cleaved caspase‐9, a marker for apoptosis through the mitochondrial pathway, was also induced by VSNL1 KD (Figure 3B). In contrast, the cleavage of caspase‐8, which is involved in the death receptor‐induced apoptosis pathway and contributes to the mitochondrial apoptosis pathway, was not augmented (Figure 3B).

We next explored effect of VSNL1 KD on the expression of the cyclin‐dependent kinase inhibitors (CDKIs), *CDKN1A* and *CDKN1B*, and the pro/antiapoptotic regulators *BAX*, *BCL2*, and *BCL2L1*. As shown in Figure S9, the mRNA levels of none of these five genes were significantly changed by the two *VSNL1* siRNAs in SW48 or SW480 cells (Figure S9).

### 3.7 Induction of apoptosis resistance by forced expression of VSNL1 in VSNL1‐negative CRC cells

**FIGURE 4:**
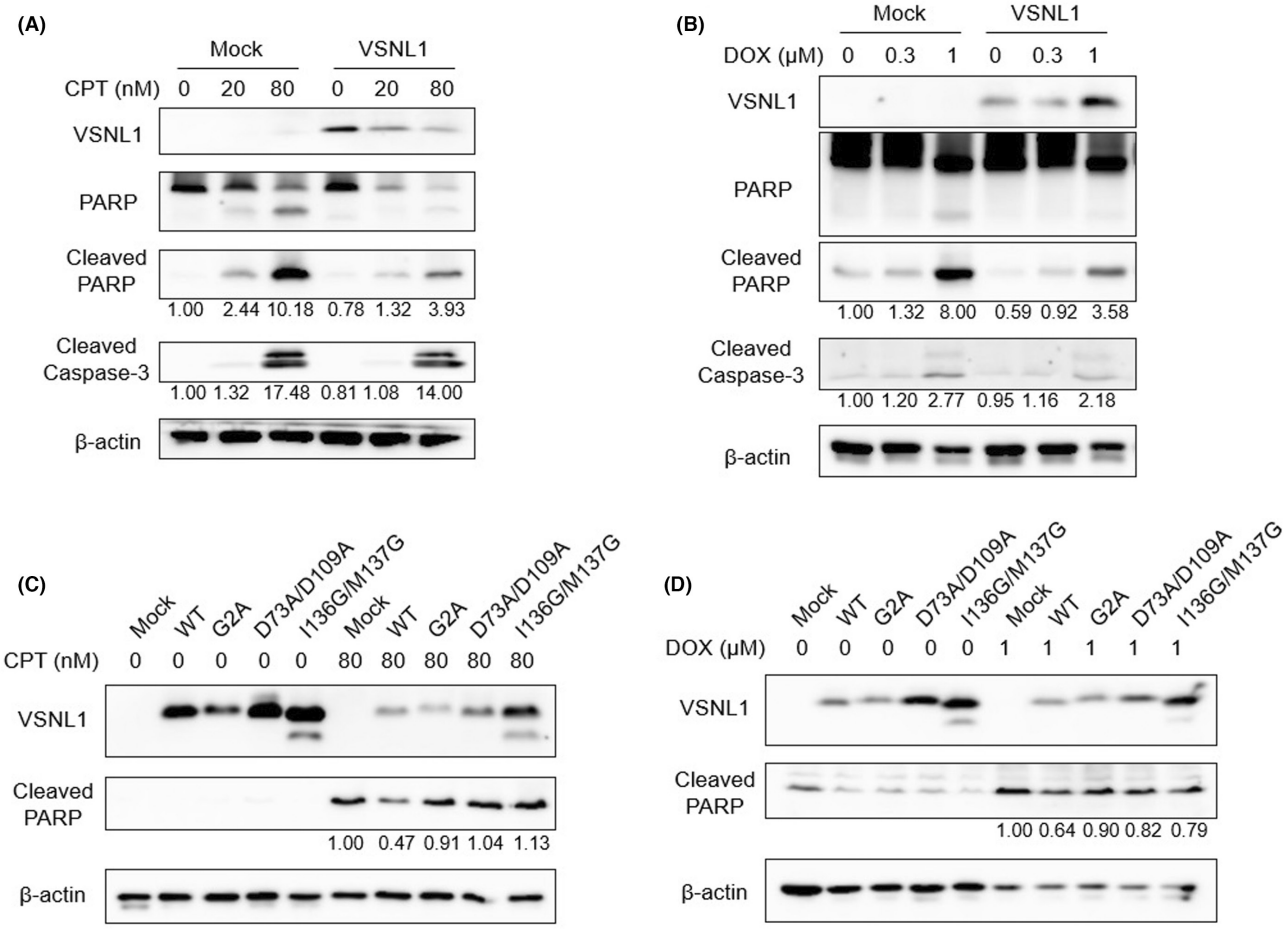
Forced expression of VSNL1 in CRC cells facilitates resistance to anticancer drug‐induced apoptosis. (A, B) Protein expression levels of cleaved PARP and cleaved caspase‐3 in RKO cells with and without forced expression of VSNL1 under treatment with camptothecin (CPT) (A) or doxorubicin (DOX) (B). (C, D) Protein expression of cleaved PARP in RKO cells expressing wild‐type (WT) VSNL1 or its mutants treated with CPT (C) or DOX (D). G2A: myristoylation‐defective mutant, D73A/D109A: Ca^2+^‐binding‐defective mutant, I136G/M137G: dimerization‐defective mutant. Band intensities were quantified using ImageJ and were normalized to β‐actin.

Because VSNL1 KD induced apoptosis in CRC cells, we hypothesized that forced expression of VSNL1 confers apoptosis resistance to VSNL1‐negative CRC cells. To test this hypothesis, we used a retroviral transduction system to stably express wild‐type VSNL1 in RKO cells, in which the Wnt/β‐catenin signaling pathway was not activated and the endogenous VSNL1 protein was not expressed (Figure 1B). Consistent with our hypothesis, the induction of PARP and caspase‐3 cleavage by the anticancer drugs camptothecin (CPT) and doxorubicin (DOX) was lower in the cells with stable expression of wild‐type VSNL1 than in control cells and this effect was dose‐dependent (Figure 4A,B). These findings suggest that VSNL1 plays an antiapoptotic role in CRC cells. By contrast, forced expression of VSNL1 did not affect Wnt/β‐catenin signaling activity or cell proliferation (Figures S8B and S10).

### 3.8 Roles of Ca^2+^‐binding, myristoylation, and dimerization of VSNL1 in apoptosis resistance

Ca^2+^‐binding, myristoylation, and dimerization are important for the function of VSNL1, which belongs to the Ca^2+^‐sensor protein family. Therefore, we investigated whether these molecular characteristics were involved in the antiapoptotic function of VSNL1. RKO cells with stable expression of wild‐type or a mutant defective in myristoylation (G2A), Ca^2+^‐binding (D73A/D109A), or dimerization (I136G/M137G), were treated with vehicles or anticancer drugs for 48 h. Then, the induction of apoptosis was evaluated using Western blotting with antibodies against cleaved PARP. The expression of cleaved PARP was lower in cells expressing wild‐type VSNL1 than in control cells after CPT or DOX treatment (Figure 4C,D). In contrast, the expression of cleaved PARP was unchanged in the cells expressing myristoylation‐, Ca^2+^‐binding‐, or dimerization‐defective VSNL1 mutants, compared with control cells, after CPT or DOX treatment (Figure 4C,D). Our findings suggest that myristoylation, Ca^2+^‐binding, and dimerization are involved in the antiapoptotic function of VSNL1 in CRC cells.

## 4 DISCUSSION

*VSNL1* expression is enhanced in CRC cells compared with normal colorectal epithelial cells.^22^ However, the molecular mechanism that underlies *VSNL1* upregulation in CRC cells is unclear. The present study revealed that *VSNL1* is a target gene of the Wnt/β‐catenin signaling pathway in CRC cells.

In a previous study, VSNL1 was downregulated by the expression of β‐catenin with gain‐of‐function mutations in the ureteric bud tips of mouse embryonic kidneys. Furthermore, forced expression of VSNL1 suppressed Wnt/β‐catenin signaling activity in mouse inner medullary collecting duct cells, suggesting that VSNL1 and the Wnt/β‐catenin signaling pathway counteract each other.^39^ In the present study, Wnt/β‐catenin signaling positively regulated the expression of *VSNL1* in CRC cells, whereas Wnt/β‐catenin signaling activity was unaffected by *VSNL1* KD or forced expression. This discrepancy in the relationship between VSNL1 and the Wnt/β‐catenin signaling pathway may be the result of tissue‐specific or developmental stage‐specific regulatory mechanisms.

In previous studies, *VSNL1* expression was frequently silenced by the methylation of its promoter region in non‐small cell lung cancers. Furthermore, the binding of nuclear respiratory factor 1 to the nuclear respiratory factor‐binding element located 50‐bp upstream of its transcription start site was important for *VSNL1* expression in non‐small cell lung cancers. In the present study, we found that the transcriptional regulation of *VSNL1* by the Wnt/β‐catenin signaling pathway was mediated via two TCF‐responsive elements in intron 1 of *VSNL1*. Although most Wnt/β‐catenin target genes are transcriptionally regulated by TCF‐responsive elements in their promoter regions, some target genes are regulated by TCF‐responsive elements in their introns or distal enhancer regions, including *RNF43* and *MYC*.^26^, ^40^, ^41^ The present study showed that *VSNL1* is one of the genes with TCF‐responsive elements in an intron.

Several datasets in the Oncomine database showed that the *VSNL1* mRNA was upregulated in CRC cells and colorectal adenoma cells. Because dysregulation of the Wnt/β‐catenin signaling pathway by *APC* inactivation is the first step in a multistep genetic model for most CRCs,^42^ VSNL1 may be involved in the initiation of colorectal carcinogenesis.

Regarding the role of VSNL1 in apoptosis, forced VSNL1 expression in a subclone of SK‐N‐AS neuroblastoma cells with low invasive ability led to the suppression of detachment‐induced apoptosis.^21^ VSNL1 KD in adrenocortical carcinoma cells (NCI‐H295R) rendered them sensitive to Ca^2+^‐induced apoptosis.^43^ In this study, we demonstrated that VSNL1 is involved in the apoptosis resistance mechanism in CRC cells. VSNL1 KD induced apoptosis in CRC cells endogenously expressing VSNL1, whereas forced expression of VSNL1 in VSNL1‐negative CRC cells conferred resistance to apoptosis induced by anticancer drugs. Although *VSNL1* is an indicator of lymph node metastasis and poor prognosis in CRC patients,^44^ *VSNL1* expression levels were not correlated with the CRC clinical stage or prognosis in the GEPIA and UALCAN databases. The effect of VSNL1 on resistance to anticancer drug‐induced apoptosis alone may not be sufficient to affect survival in CRC patients.

Although VSNL1 is involved in tumorigenesis in various types of cancer,^17^, ^18^, ^19^, ^20^, ^21^, ^22^, ^43^, ^45^, ^46^ the significance of myristoylation, Ca^2+^‐binding, or dimerization has not been investigated. The present study showed that the ability of VSNL1 to suppress anticancer drug‐induced apoptosis depended on myristoylation, Ca^2+^‐binding, and protein dimerization, thus suggesting that the Ca^2+^‐myristoyl switch is indispensable for the antiapoptotic function of VSNL1 in CRC cells.

Although we showed that VSNL1 is involved in apoptosis of CRC cells via the Ca^2+^‐myristoyl switch, we did not identify proapoptotic or antiapoptotic regulators downstream of VSNL1. Further studies including gene expression profile analyses of cells with KD or forced expression of VSNL1 are needed to identify downstream regulators of VSNL1‐associated apoptosis.

There are limitations to this study that should be noted. First, because VSNL1 KD was performed only transiently using siRNA, the effect of VSNL1 suppression on proliferation, migration, or invasion could not be investigated. In addition, in vivo experiments such as subcutaneous transplantation in immunodeficient mice were not performed. Studies using cells with stable KD or KO of VSNL1 are required to elucidate its role in colorectal tumorigenesis.

In conclusion, *VSNL1* is a novel target gene of the Wnt/β‐catenin signaling pathway and contributes to apoptosis resistance via the Ca^2+^‐myristoyl switch in CRC cells.

## AUTHOR CONTRIBUTIONS

**Hiroki Tage:** Conceptualization (equal); data curation (equal); investigation (lead); methodology (equal); validation (equal); writing – original draft (equal). **Kiyoshi Yamaguchi:** Conceptualization (supporting); funding acquisition (equal); investigation (supporting); methodology (equal); supervision (supporting). **Saya Nakagawa:** Investigation (supporting); methodology (supporting). **So Kasuga:** Investigation (supporting). **Kiyoko Takane:** Investigation (supporting); supervision (supporting). **Yoichi Furukawa:** Conceptualization (supporting); project administration (supporting); supervision (supporting); writing – original draft (supporting). **Tsuneo Ikenoue:** Conceptualization (lead); data curation (equal); investigation (lead); supervision (lead); validation (lead); writing – original draft (lead).

## CONFLICT OF INTEREST STATEMENT

The authors have no conflict of interest to disclose.

## Supporting information


Data S1.

## Data Availability

All relevant data are available from the authors upon request.
