# Supplementary material for: Visinin‐like 1, a novel target gene of the Wnt/β‐catenin signaling pathway, is involved in apoptosis resistance in colorectal cancer
Source: Cancer Med. 2023 Apr 25;12(12):13426–37. doi: 10.1002/cam4.5970 (PMC10315817; doi:10.1002/cam4.5970)
Supplement: Supplementary file 1 — Data S1. [file CAM4-12-13426-s001.pdf]

Supplementary Figure S1. Disruption of *APC* gene in HAP1 cells.

A

Name of clones	DNA change	Protein change
APC-KO #1	c.del319_321	p.delSer107
APC-KO #2	c.del2569_2570	p.Gly857Asnfs*54
APC-KO #3	c.del2558_2576	p.Glu853Valfs*2

B

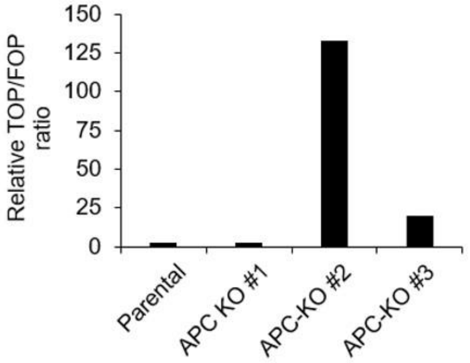

C

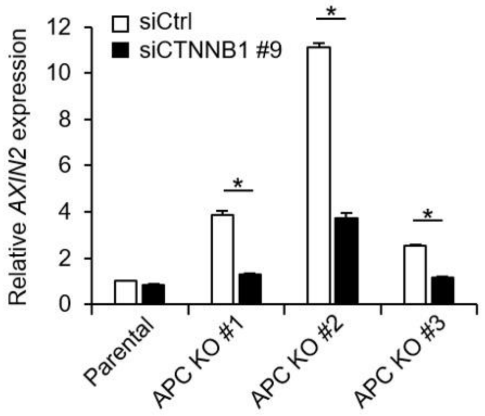

Supplementary Figure S2. Regulation of VSNL1 by Wnt/β-catenin signaling in HAP1 cells

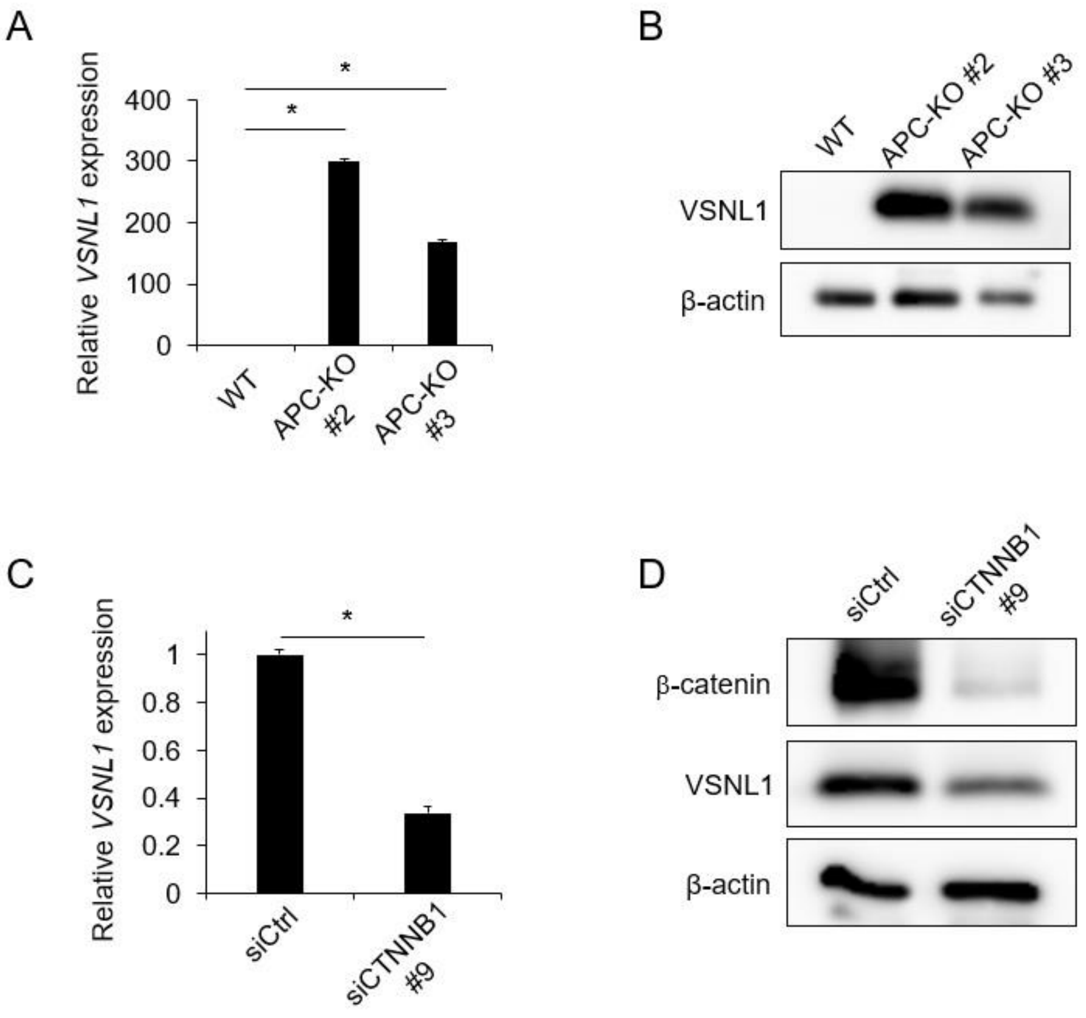

Supplementary Figure S3. Relationship of mRNA expression levels of *VSNL1* in human CRC and normal colon tissue samples.

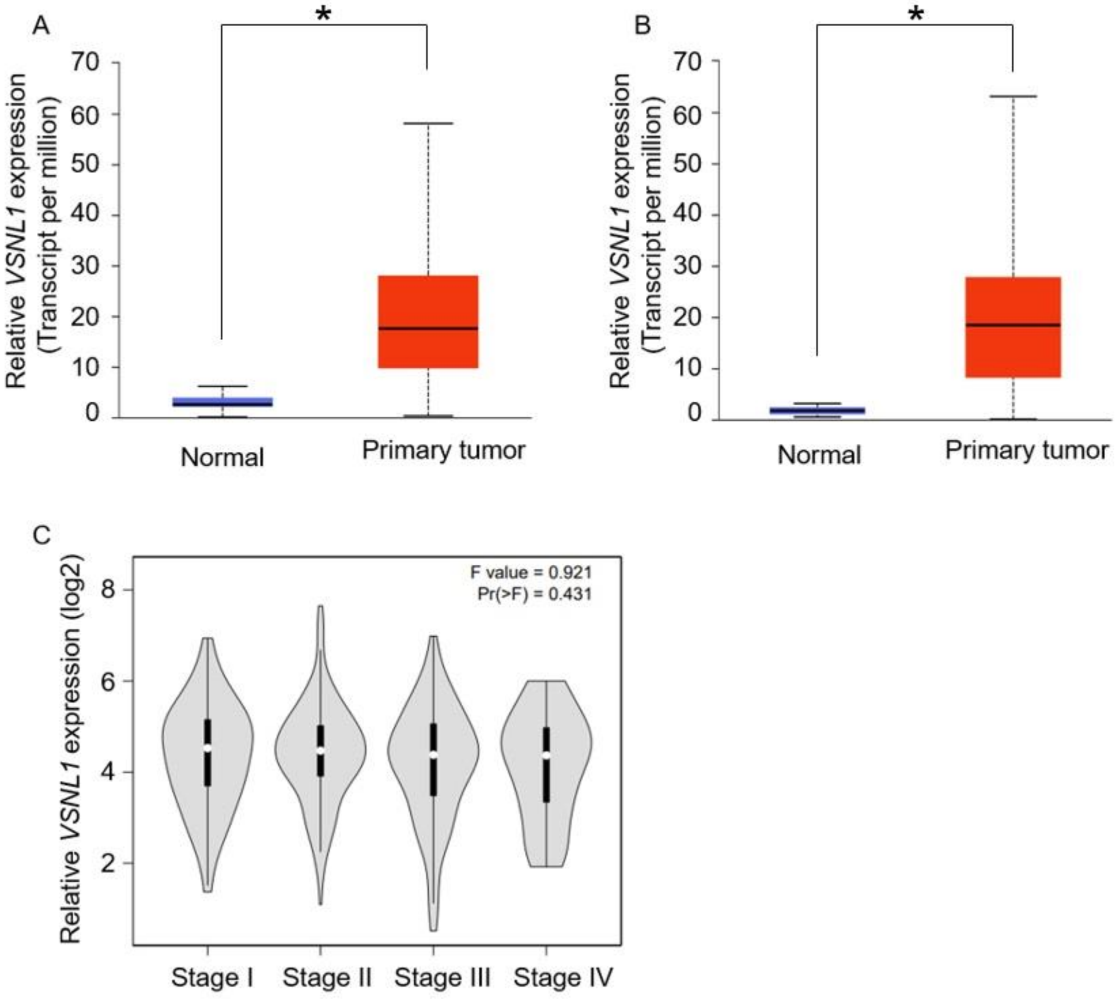

Supplementary Figure S4. Relationship of mRNA expression levels of *VSNL1* in human colorectal adenoma and normal colon tissue samples.

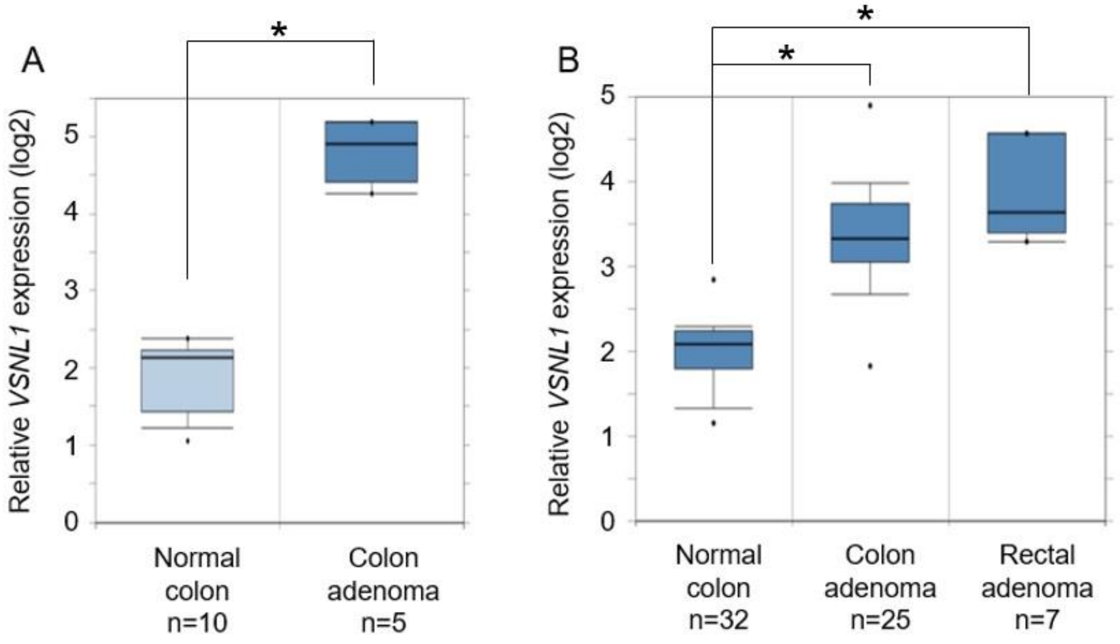

Supplementary Figure S5. Relationship of mRNA expression levels between *VSNL1* and *LGR5*, *CD44*, or *AXIN2* in human CRC samples.

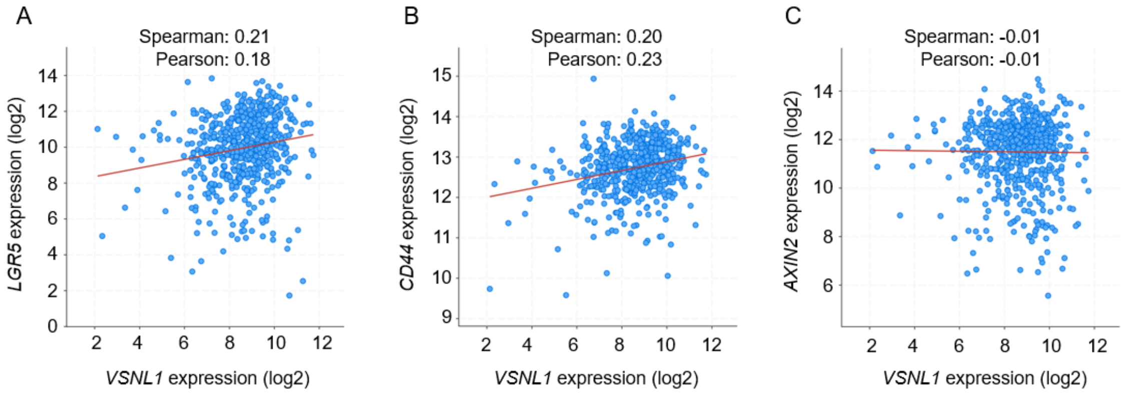

Supplementary Figure S6. Effect of β-catenin knockdown on *VSNL1* promoter activity in CRC cell lines

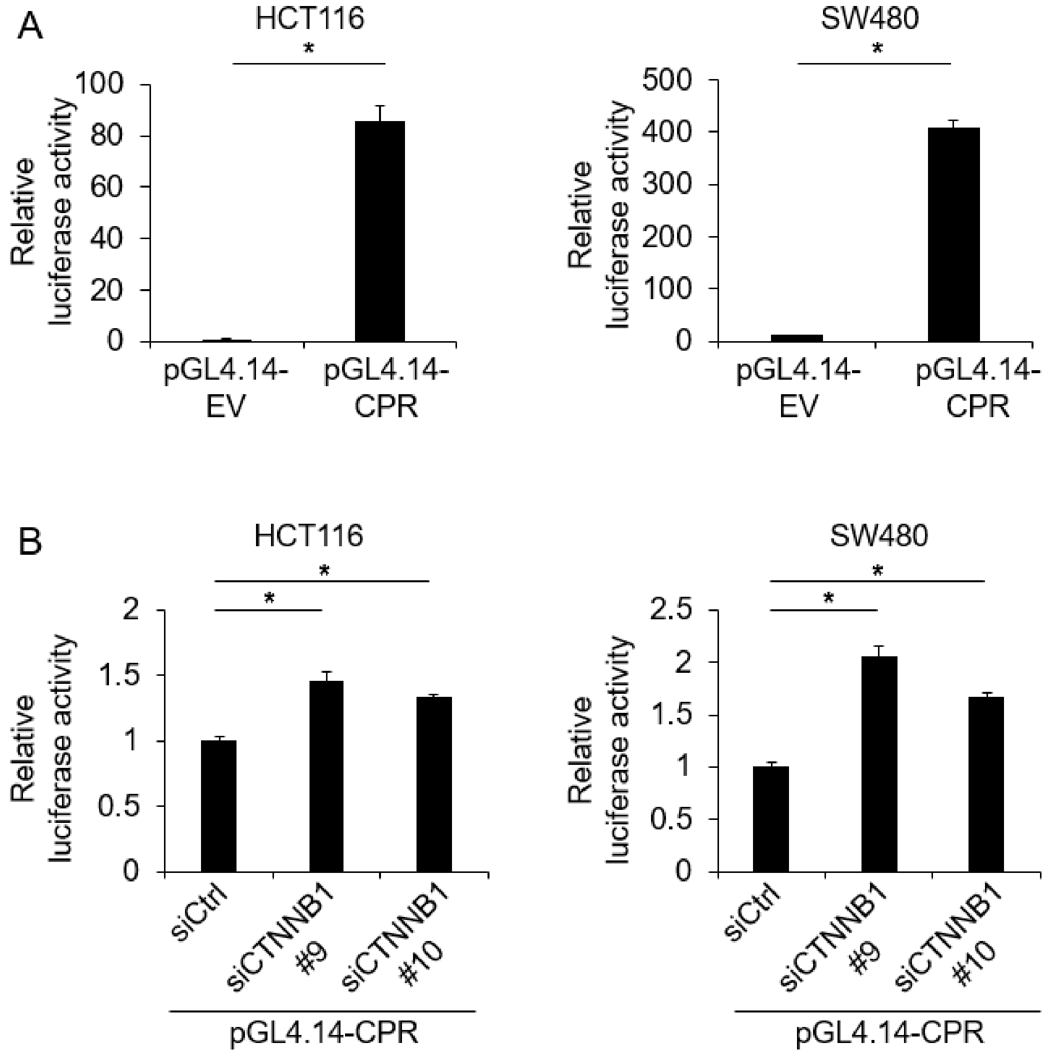

Supplementary Figure S7. Effect of β-catenin knockdown on the reporter activity of the TBE1/3 mutant plasmid in CRC cell lines.

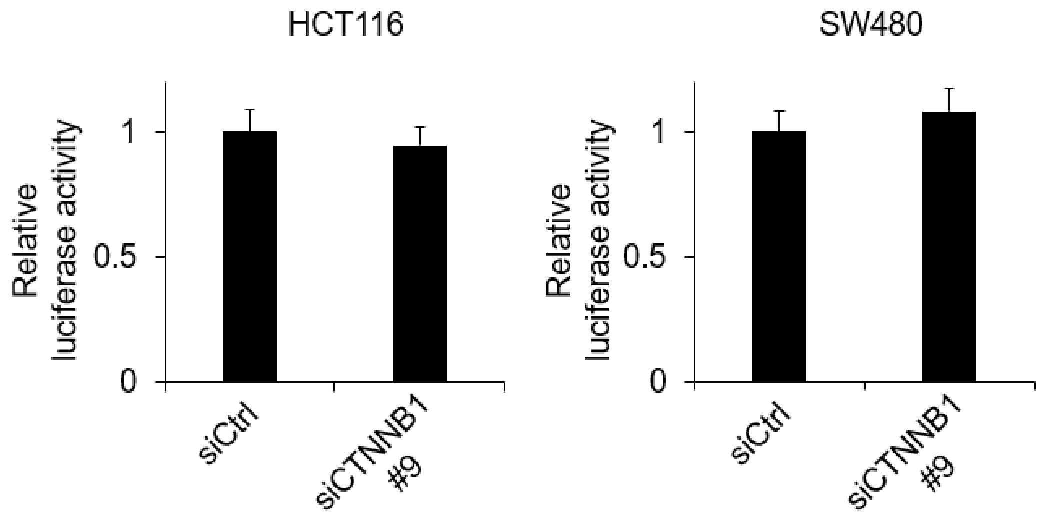

Supplementary Figure S8. Effect of KD or forced expression of VSNL1 on Wnt/β-catenin signaling in CRC cell lines.

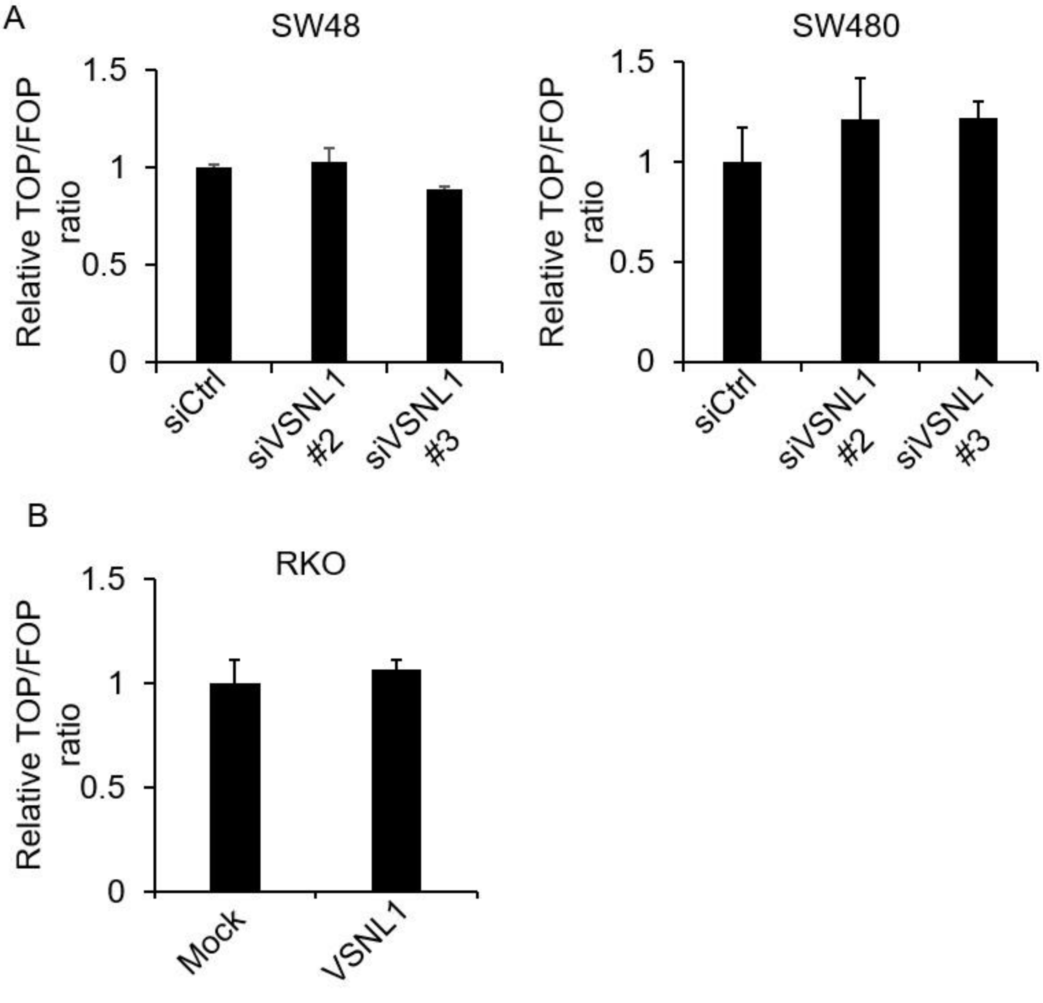

Supplementary Figure S9. Effect of VSNL1 KD on the expression of CDK inhibitors and pro/antiapoptotic regulators.

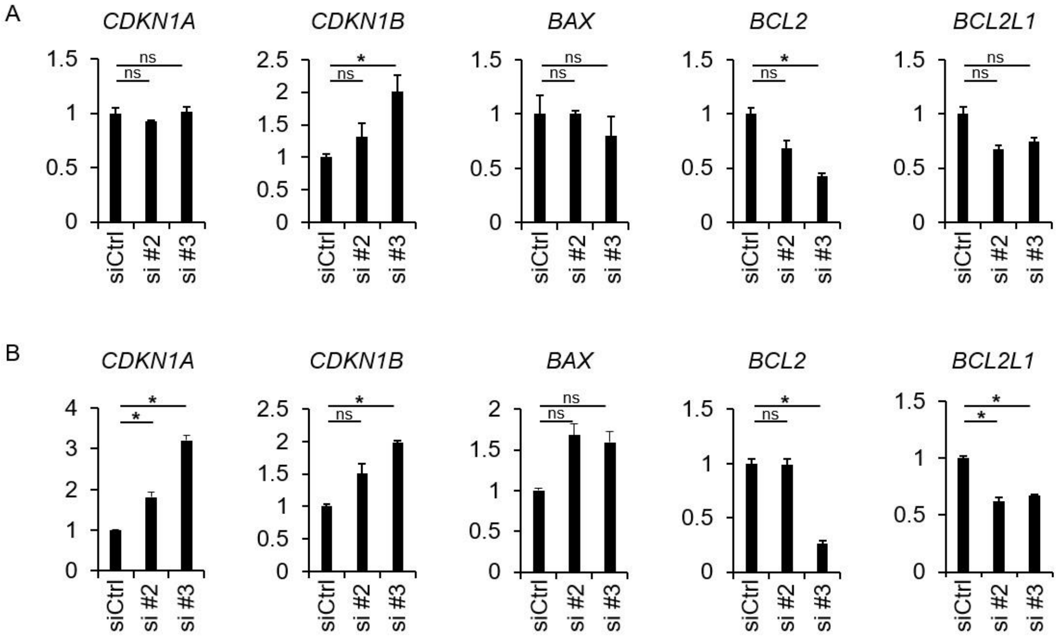

Supplementary Figure S10. Effect of forced expression of VSNL1 on proliferation of RKO cells.

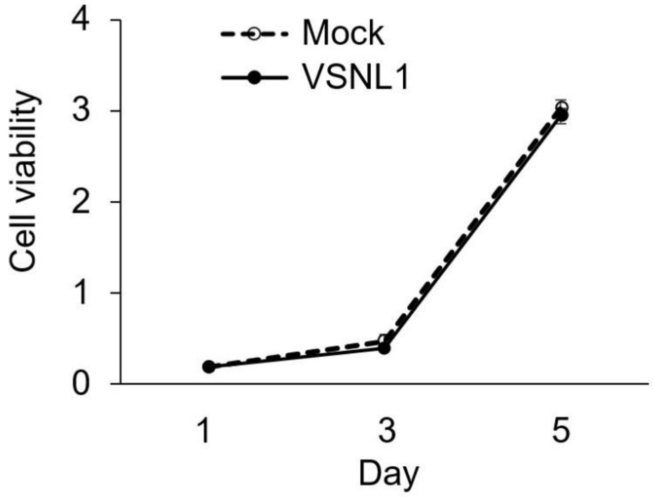

Supplementary Figure S1. Disruption of *APC* gene in HAP1 cells.

(A) Changes of DNA and protein sequences in HAP1-APC-KO clones (#1-3). (B) Relative Wnt/β-catenin-dependent transcriptional activity of HAP1-APC-KO clones. A TOPFLASH/FOPFLASH (TOP/FOP) assay was performed to determine the Wnt/β-catenin-dependent transcriptional activity in parental HAP1 cells and HAP1-APC-KO clones (#1-3). (C) Relative expression levels of *AXIN2* mRNA in parental HAP1 cells and APC-KO clones (#1-3) treated with control siRNA (siCtrl) and *CTNNB1* siRNA #9 (siCTNNB1 #9) for 48 h.

Supplementary Figure S2. Regulation of VSNL1 by Wnt/β-catenin signaling in HAP1 cells.

(A) Relative expression levels of *VSNL1* mRNA in parental HAP1 cells (WT) and APC-KO HAP1 clones (APC-KO #2 and #3). (B) VSNL1 protein expression in parental HAP1 cells (WT) and APC-KO HAP1 clones (APC-KO #2 and #3). (C) Relative expression levels of *VSNL1* mRNA in HAP1-APC-KO cells treated with control siRNA (siCtrl) and *CTNNB1* siRNA #9 (siCTNNB1 #9) for 48 h. (D) VSNL1 protein expression in HAP1-APC-KO cells treated with control siRNA (siCtrl) and

CTNNB1 siRNA #9 (siCTNNB1 #9) for 48 h. Data are presented as means ± SEs of at least three independent experiments.

Supplementary Figure S3. Relationship of mRNA expression levels of *VSNL1* in human CRC and normal colon tissue samples.

(A, B) *VSNL1* mRNA expression in CRC and normal colorectal tissues. The data were obtained from the TCGA dataset in UALCAN. (A) Colon adenocarcinoma vs normal colon tissue. (B) Rectal adenocarcinoma vs normal rectal tissue. (C) VSNL1 mRNA expression in CRC at different clinical stages. The data were obtained from the TCGA dataset in GEPIA. The significance of differences was estimated by Student's *t*-test (A, B).

Supplementary Figure S4. Relationship of mRNA expression levels of *VSNL1* in human colorectal adenoma and normal colon tissue samples.

The data were obtained from the Skrzypczak colorectal dataset (A) and Sabates-Bellver Colon dataset (B) in Oncomine. The significance of differences was estimated by Student's *t*-test.

Supplementary Figure S5. Relationship of mRNA expression levels between *VSNL1* and *LGR5*, *CDC44*, or *AXIN2* in CRC samples.

Dataset of mRNA expression levels of *VSNL1*, *LGR5*, *CD44*, and *AXIN2* in 594 CRC samples was obtained from TCGA PanCancer Atlas. Quantification of the mRNA expression level of each gene was performed using RNA-seq by Expectation Maximization (RSEM) (batch normalized from Illumina HiSeq_RNASeqV2).

Supplementary Figure S6. Effect of β-catenin knockdown on *VSNL1* promoter activity in CRC cell lines.

(A) Transcriptional activity of candidate promoter region (CPR) of *VSNL1* that encompasses a 1.2-kb region (-957 bp to +211 bp from the TSS) was measured using a reporter plasmid containing the CPR (pGL4.14-CPR) and an empty vector (pGL4.14-EV). (B) Effect of β-catenin KD on the promoter activity of *VSNL1*. A reporter assay was performed using a reporter plasmid containing the CPR (pGL4.14-CPR) in combination with control siRNA or *CTNNB1* siRNAs (#9 and #10) in HCT116 and SW480 cell lines.

Supplementary Figure S7. Effect of β-catenin knockdown on the reporter activity of the TBE1/3 mutant plasmid in CRC cell lines.

A reporter assay was performed using reporter plasmids containing the TBE1 and TBE3 mutations in combination with control siRNA or *CTNNB1* siRNA #9 in HCT116 and SW480 cells.

Supplementary Figure S8. Effect of KD or forced expression of VSNL1 on Wnt/β-catenin signaling in CRC cell lines.

A TOP/FOP assay was performed to determine Wnt/β-catenin-dependent transcriptional activity in SW48 (A) and SW480 (B) cells treated with a control siRNA or *VSNL1* siRNA, and RKO (C) cells with and without forced expression of VSNL1.

Supplementary Figure S9. Effect of KD or forced expression of VSNL1 on the expression of CDK inhibitors and pro/antiapoptotic regulators.

Relative mRNA levels of *CDKN1A*, *CDKN1B*, *BAX*, *BCL2*, and *BCL2L1* in SW48 (A) and SW480 (B) cells transfected with control siRNA or two siRNAs for VSNL1 as determined by RT-qPCR.

Supplementary Figure S10. Effect of forced expression of VSNL1 on proliferation of RKO cells.

Proliferation of RKO cells with and without forced expression of VSNL1 as determined by WST-8 assay.

Supplementary Table 1. Sequence of gRNAs, siRNAs, and primers

Sequence of Oligos used for constructing gRNA expression vectors for *APC* disruption

Name	Strand	Sequence (5'>3')
APC-544	Sense	CACCGGGATCTGTATCAAGCCGTTC
	Antisense	AAACGAACGGCTTGATACAGATCCC
APC-2713	Sense	CACCGTTGGAGAGAGAACGCGGAAT
	Antisense	AAACATTCCGCGTTCTCTCTCCAAC

Sequence of siRNA

Name	Sequence (5'>3')
siCTNNB1 #9	GAUCCUAGCUAUCGUUCUU
siCTNNB1 #10	UAAUGAGGACCUAUACUUA
siVSNL1 #2	GUAUUACUUCUGCAGUGCGACAUCC
siVSNL1 #3	GCAUGAACUCAAGCAGUGGUACAAA

Sequence of primers used in RT-qPCR

Name	Strand	Sequence (5'>3')
VSNL1	Forward	TGGATGGTGATGGCAACAT
	Reverse	TGCCTACCATTTTGTAGATAGCC
AXIN2	Forward	CCACACCCTTCTCCAATCC
	Reverse	TGCCAGTTTCTTTGGCTCTT
BCL2	Forward	ACCTGGATCCAGGATAACG
	Reverse	CTGGGGCCGTACAGTTCC
BCL2L1	Forward	AGCGTAGACAAGGAGATGC
	Reverse	GCTGCTGCATTGTTCCCATA
BAX	Forward	ATGTTTTCTGACGGCAACTTC
	Reverse	ATCAGTTCCGGCACCTTG
CDKN1A	Forward	CCGAAGTCAGTTCCTTGTGG
	Reverse	CATGGGTTCTGACGGACAT
CDKN1B	Forward	TAGCGGAGCAATGCGCAGGA
	Reverse	AACCGGCATTTGGGGAACCGT
HPRT1	Forward	TGATAGATCCATTCCTATGACTGTAGA
	Reverse	CAAGACATTCTTTCCAGTTAAAGTTG
GAPDH	Forward	AGCCACATCGCTCAGACAC
	Reverse	GCCCAATACGACCAAATCC

Sequence of primers used in ChIP-qPCR

Name	Strand	Sequence (5'>3')
VSNL1	Forward	AGAGCCACTCCCTTTACAAAC
	Reverse	ACCCAGAGCTAAATAGAATGAGC
RNF43	Forward	TCAACTCTCTGGATAAGGTGGAATAGC
	Reverse	GACTTTTGGGGTGGGTGGGAAATA
GAPDH	Forward	AGCTCAGGCCTCAAGACCTT
	Reverse	AAGAAGATGCGGCTGACTGT

Sequence of primers in cloning *VSNL1* -CER and CPR

Name	Strand	Sequence (5'>3')
VSNL1 -CPR	Forward	AAGCTCGAGACCAAACATTTATTGGGCAC
	Reverse	TACAGATCTGCGCACGGTCACTTAAC
VSNL1 -CER	Forward	TAGCTCGAGTGCTTTAGGAAAGGTGTGGTCC
	Reverse	TCAAGATCTGGTTCTCCTGAGCTTCTCCT

Sequence of primers used in site-directed mutagenesis for TBE mutants

Name	Strand	Sequence (5'>3')
TBE1 mut	Forward	TCCAATGAACTTATCGCAGCACCGAAAACCC
	Reverse	GGGTTTTCGGTGCTGCGATAAGTTCATTGGA
TBE2 mut	Forward	AGGGCCCTGGGATGAGCGAATCCTGGAAAAG
	Reverse	CTTTTCCAGGATTCGCTCATCCCAGGGCCCT
TBE3 mut	Forward	CTTTTAGGATTCACAGGTAGATCAGCGACCTTTAGTTCTTAGGCT
	Reverse	AGCCTAAGAACTAAAGGTCGCTGATCTACCTGTGAATCCTAAAAG

Supplementary Table 1. Sequence of gRNAs, siRNAs, and primers (continued)

Sequence of primers used in site-directed mutagenesis for VSNL1 mutants

Name	Strand	Sequence (5'>3')
G2A	Forward	ATGGCGAAGCAGAATAGCAAACTGGCCCC
	Reverse	GGGGCCAGTTTGCTATTCTGCTTCGCCAT
D73A	Forward	CCTTCCGAACCTTCGCCAAGAATGGGGACGG
	Reverse	CCGTCCCCATTCTTGGCGAAGGTTCGGAAGG
D109A	Forward	CCTTCAATATGTATGCCCTGGATGGTGATGG
	Reverse	CCATCACCATCCAGGGCATACATATTGAAGG
I136G/M137G	Forward	ATGGTAGGCACTGTGGGCGGGATGAAAATGAATGA
	Reverse	TCATTCATTTTCATCCCGCCCACAGTGCCTACCAT

Supplementary Table 2. List of 64 candidate target genes of Wnt/β-catenin signaling

Gene	FC ([APC-KO #2] vs [WT])	FC ([APC-KO #3] vs [WT])	FC ([siCTNNB1] vs [siCtrl])
C2orf83	5921.1	112.2	-51.9
DKK2	680.2	224.5	-14.5
PLSCR5	7640.1	20380.1	-12.5
APCDD1	832.2	2356.5	-11.7
PTX3	2531.0	8704.5	-9.0
CDKN1C	2.7	6.9	-7.8
TRPM3	2220.3	11849.4	-7.6
NKD1	9.2	59.7	-6.8
CTCFL	30.7	19.8	-6.8
STAT4	361.4	769.5	-6.5
CCL2	9.0	62.3	-5.9
FOXL1	6.0	9.6	-5.6
DNM3	22.5	42.8	-5.3
PDE5A	5.1	21.6	-5.2
AXIN2	11.3	23.5	-5.2
DISC1	3.2	7.7	-5.1
TNFAIP8L3	2.9	15.1	-4.7
MAOB	1754.3	11226.0	-4.7
DIO2	64.0	31.1	-4.3
RNF43	40.2	67.7	-4.3
ADRA2C	5.7	8.6	-4.1
CLSTN2	542.6	670.8	-4.0
LPPR4	26.5	224.4	-4.0

HS3ST3A1	8.9	11.9	-3.9
VSNL1	119.6	208.4	-3.8
GAD1	21.7	9.9	-3.7
PLSCR1	4.6	8.2	-3.6
CEACAM1	170.2	460.2	-3.6
ZNF385A	4.1	4.7	-3.5
SH3GL2	10.6	28.2	-3.3
ANKRD37	4.8	15.5	-3.3
AHR	8.3	25.6	-3.1
NEFM	28.7	18.2	-3.1
TMPRSS15	63.9	930.4	-3.0
SEMA3C	281.7	265.6	-3.0
TRDN	7799.0	4226.0	-3.0
CASZ1	7.4	8.9	-3.0
MSX2	2.7	6.5	-2.9
STOX1	2.2	7.5	-2.9
KANK4	42.0	112.1	-2.8
AIMP1	2.8	4.2	-2.8
MYH7B	20.7	47.1	-2.8
TLL1	12.1	24.2	-2.7
ZNF703	3.5	5.9	-2.7
IL33	1514.3	1275.4	-2.7
GRB14	6.3	4.2	-2.7
TBX2	3.1	5.3	-2.7
KREMEN1	3.4	4.9	-2.6

NEFL	343.5	2753.9	-2.6
ELF1	11.4	12.1	-2.5
PCDH17	5.9	4.7	-2.5
CXADR	4.4	4.7	-2.4
LEPR	10.5	22.5	-2.4
BAMBI	4.6	14.3	-2.4
RHOB	5.7	2.9	-2.3
SESN3	2.3	2.3	-2.2
TNFRSF19	53.6	49.8	-2.1
SFRP1	3.4	3.9	-2.1
STOX2	6.3	6.8	-2.1
PDE3A	165.7	6917.1	-2.1
ZMAT4	114.8	256.8	-2.1
PLA2G2A	7955.0	72318.4	-2.1
ZPLD1	1321.1	77846.1	-2.0
CA5B	10.6	3.7	-2.0

Supplementary Table 3. KEGG pathway analysis of 64 genes by MSigDB

Gene Set Name	Description	Overlapped gene symbols	p-value	FDR q-value
KEGG_WNT_SIGNALING_PATHWAY	Wnt signaling pathway	*AXIN2*<br>*DKK2*<br>*SFRP1*<br>*NKD1*	9.07E-05	1.69E-02
